# Evolutionary history of the recruitment of conserved developmental genes in association to the formation and diversification of a novel trait

**DOI:** 10.1186/1471-2148-12-21

**Published:** 2012-02-15

**Authors:** Leila T Shirai, Suzanne V Saenko, Roberto A Keller, Maria A Jerónimo, Paul M Brakefield, Henri Descimon, Niklas Wahlberg, Patrícia Beldade

**Affiliations:** 1 Instituto Gulbenkian de Ciência, Rua da Quinta Grande 6, P-2780-156 Oeiras, Portugal; 2 Institute of Biology, Leiden University, Sylviusweg 72, 2333 BE Leiden, The Netherlands; 3 Université de Provence, 3 Place Victor Hugo, F-13331, Marseille CEDEX 03, France; 4 Department of Biology, University of Turku, 20014 Turku, Finland; 5 Department of Genetics and Evolution, University of Geneva, Quai Ernest-Ansermet 30, 1211 Geneva, Switzerland; 6 Department of Zoology, Cambridge University, Downing Street, Cambridge CB2 3EJ, UK; 7 2 Boulevard Rougemont, F-13012 Marseille, France

## Abstract

**Background:**

The origin and modification of novel traits are important aspects of biological diversification. Studies combining concepts and approaches of developmental genetics and evolutionary biology have uncovered many examples of the recruitment, or co-option, of genes conserved across lineages for the formation of novel, lineage-restricted traits. However, little is known about the evolutionary history of the recruitment of those genes, and of the relationship between them -for example, whether the co-option involves whole or parts of existing networks, or whether it occurs by redeployment of individual genes with *de novo *rewiring. We use a model novel trait, color pattern elements on butterfly wings called eyespots, to explore these questions. Eyespots have greatly diversified under natural and sexual selection, and their formation involves genetic circuitries shared across insects.

**Results:**

We investigated the evolutionary history of the recruitment and co-recruitment of four conserved transcription regulators to the larval wing disc region where circular pattern elements develop. The co-localization of Antennapedia, Notch, Distal-less, and Spalt with presumptive (eye)spot organizers was examined in 13 butterfly species, providing the largest comparative dataset available for the system. We found variation between families, between subfamilies, and between tribes. Phylogenetic reconstructions by parsimony and maximum likelihood methods revealed an unambiguous evolutionary history only for Antennapedia, with a resolved single origin of eyespot-associated expression, and many homoplastic events for Notch, Distal-less, and Spalt. The flexibility in the (co-)recruitment of the targeted genes includes cases where different gene combinations are associated with morphologically similar eyespots, as well as cases where identical protein combinations are associated with very different phenotypes.

**Conclusions:**

The evolutionary history of gene (co-)recruitment is consistent with both divergence from a recruited putative ancestral network, and with independent co-option of individual genes. The diversity in the combinations of genes expressed in association with eyespot formation does not parallel diversity in characteristics of the adult phenotype. We discuss these results in the context of inferring homology. Our study underscores the importance of widening the representation of phylogenetic, morphological, and genetic diversity in order to establish general principles about the mechanisms behind the evolution of novel traits.

## Background

The origin and diversification of novel traits are central and longstanding issues in evolutionary biology [1]. Evolutionary novelties are lineage-restricted traits often associated with new adaptive functions [1,2]. Compelling examples include angiosperm flowers, beetle horns, bird feathers, and butterfly wing color patterns. Studies in evolutionary developmental biology have shown that the origin of novel traits often involves the recruitment, or co-option, of conserved genetic circuitries. This idea is captured in the expression "teaching old genes new tricks" [3], used to explain the genetic mechanisms through which novel traits arise.

The "new tricks" learnt by the "old genes" can involve different, non-mutually exclusive mechanisms (see [4-8]), such as the acquisition of novel expression domains (*e.g*. the Hox gene *Antennapedia *in butterfly eyespots [9]), of novel regulators (*e.g. homothorax *in beetle horns [10]), and of novel downstream targets (*e.g*. Engrailed regulation of *yellow *in *Drosophila *wing spots [11]). Despite the growing body of knowledge on the redeployment of shared genes for the development of lineage-restricted traits, key questions remain unanswered. For example, are entire pathways recruited as a whole or are individual genes co-opted and re-wired *de novo *[12]? How do recruited or rebuilt pathways diversify along with trait diversification? Widening the representation of both phylogenetic and morphological diversity, together with focus on genetic networks rather than single genes, will be crucial to solving these issues (see [13]). In this study, we provide a taxonomically and genetically wide survey of a model evolutionary novelty, butterfly eyespots, to investigate the origin and diversification of the genetic circuitry associated to its development.

Eyespots are wing pattern elements composed of concentric rings of different colors, found in several lepidopteran species. They are involved in mate choice [14,15] and predator avoidance [16,17], and their diversification is shaped by natural and sexual selection (see [18]). Eyespots are one of the distinct types of pattern elements recognized in the "Nymphalid Groundplan" [19-21]. Based on morphology and position of pattern elements, this Groundplan summarizes homologies across butterflies from the family Nymphalidae [22]. Series of eyespots, or border ocelli, run marginally along the antero-posterior wing axis of most nymphalids, sometimes showing dramatic variation both within and between species (*e.g*. in the color and the number of different rings [20,23]). At the same time, non-nymphalid species (for example, of the family Papilionidae) can also have circular pattern elements whose morphology resembles that of nymphalid eyespots to different extents [23-25], even when not in equivalent positions of the wing (*cf*. the conserved venation pattern). In order to cover the diversity in morphology and in position of eyespots *s.s*. (*i.e*., border ocelli) and eyespot-like circular pattern elements - hereafter referred to as "(eye)spots" to encompass all diversity, we assayed a number of species across three butterfly families. This broad phylogenetic coverage of phenotypic diversity is presented along with data on the putative genetic circuitry associated to early eyespot specification.

Butterfly eyespots provide a good illustration of the recruitment of genetic circuitry implicated in developmental processes shared by all insects for the formation of novel traits. This includes commonalities between eyespot development (exclusive of butterflies) and processes such as embryonic development [26,27], appendage formation [28,29], and wound healing [27,30] (conserved across insects). The colored rings that make up eyespots are sequentially formed in pupal wings [31,32], around organizing centers which are themselves specified earlier in larval wing discs (reviewed in [33]). Recently, examination of the expression of conserved genes *Antennapedia *(*Antp*), *Notch *(*N*), and *Distal-less *(*Dll*) during the initial stages of organizer establishment revealed intriguing differences among lineages within nymphalids [9,34]. However, the lack of gene expression data outside this clade prevented the reconstruction of the evolutionary history of the recruitment of those genes for expression in larval eyespot fields. Here, we increased the taxonomic sampling by including representatives of an additional nymphalid clade and two non-nymphalid families. We also examined the expression of another transcription factor in the presumptive organizer, Spalt (Sal) [30], in all species sampled. Phylogenetic analysis of this comprehensive dataset revealed great flexibility in which genes (and combinations of genes) are expressed in association with this novel trait in different lineages.

## Results and Discussion

**Figure 1:**
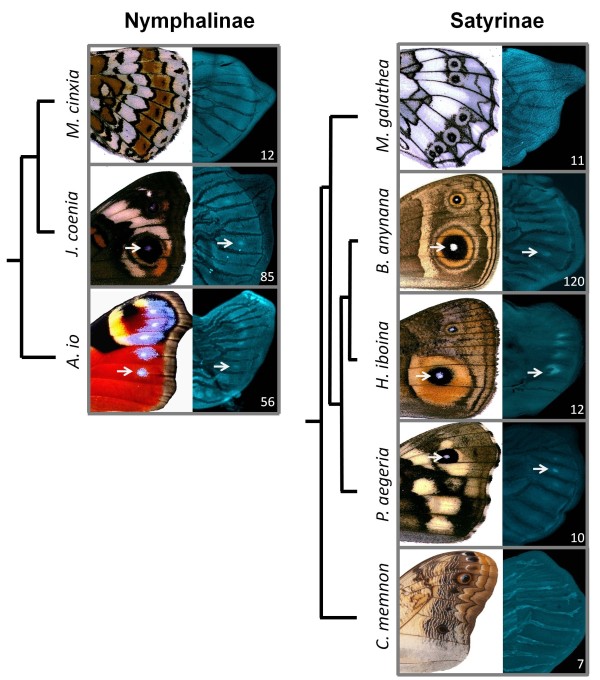
**Localization of transcription factor Sal in presumptive nymphalid eyespot organizers**. Immunostainings of Sal protein in last-instar larval wings of Nymphalinae and Satyrinae species with the corresponding adult wing (left) and sample size (bottom right corner). *Sal *expression in presumptive eyespot centers starts after tracheal expansion into the vein lacunae (corresponding to stages 0.75-1.25 *cf*. [37]). The individual wings shown here are developmental stage ~2. White arrows provide reference for the location of presumptive organizers of eyespot development. When expression is absent in forewings, it is also undetected on the hindwings.

**Figure 2:**
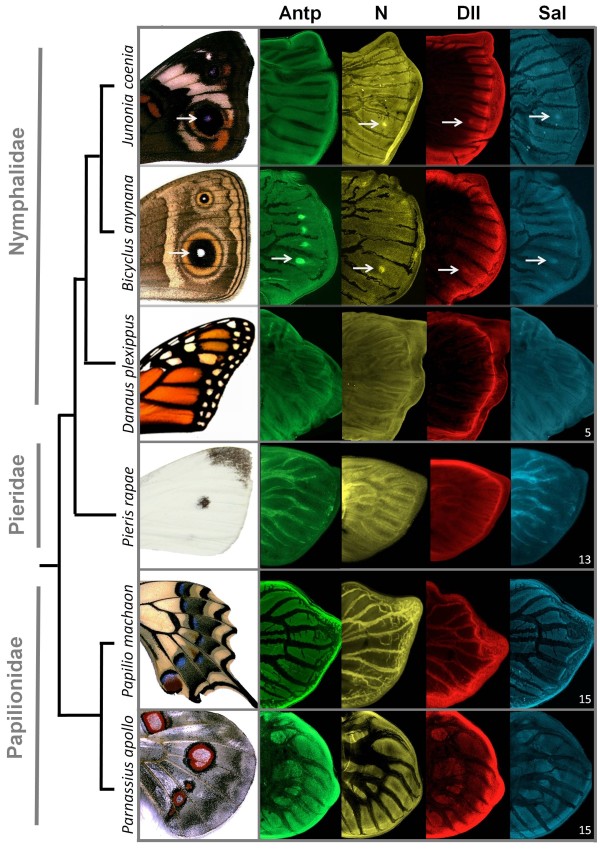
**Localization of four developmental proteins in presumptive (eye)spots of outgroup species**. Detection of Antp (green), N (yellow), Dll (red), and Sal (blue) proteins for outgroup species *D. plexippus *(Nymphalidae, Danainae), *P. rapae *(Pieridae), and *P. machaon *and *P. apollo *(Papilionidae) with the adult wing (left) and sample size (bottom right corner). *J. coenia *(Nymphalinae) and *B. anynana *(Satyrinae) expression patterns are shown as reference for respective subfamilies (*cf*. [9] and Figure 1). Note that, in some images, the localization of the eyespot organizer genes at the center of a wing compartment bordered by veins in larval wings does not associate to any eyespot in the adult wings. In these instances, the expression of such genes disappears during eyespot development but it reflects the potential of those compartments to form an eyespot (as it happens in some genetic stocks; see [36,38]).

**Figure 3:**
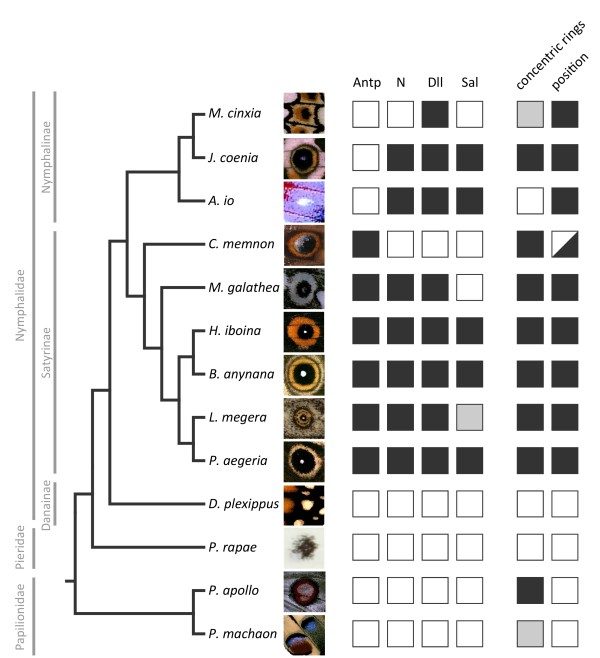
**Summary of expression data for the four developmental genes and adult (eye)spot traits**. Complete dataset of the four transcription regulators targeted in this study for all 13 species coded as the presence (black) or absence (white) of expression (*Lasiommata megera*'s *Sal *expression could not be determined, grey box). Data for Nymphalidae *Antp, N*, and *Dll *expression were obtained from [9], *Sal *expression for Nymphalidae is presented in Figure 1, and expression of all genes for outgroup species in Figure 2. Representative (eye)spots are illustrated on the right of the species name, and their phenotype is coded for characteristic aspects of Nymphalid border ocelli: 1) "concentric rings" relates to the occurrence of multiple concentric rings (black), of non-concentric rings (grey), and of a single spot (white); and 2) "position" relates to (eye)spot localization in the distal region of the wing *cf*. the Nymphalid Groundplan (black), versus in other regions (white). Notice that *C. memnon *bears eyespots in both positions (see Figure 5).

**Figure 4:**
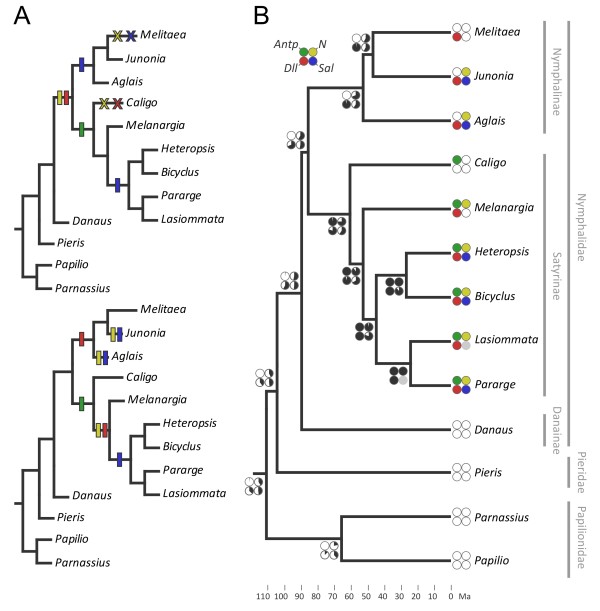
**Ancestral reconstruction of protein recruitment to presumptive eyespot center**. Parsimony (**A**) and maximum likelihood (**B**) reconstructions of the evolutionary history of the recruitment of Antp, N, Dll, and Sal for expression at the putative (eye)spot organizers. (A) Two equally parsimonious scenarios with different optimizations are shown: ACCTRAN favoring reversals (top), and DELTRAN favoring parallelisms (bottom). Hash marks represent gain and × loss of expression. (B) The estimated probabilities for each protein at the presumptive (eye)spot centers is represented by piecharts at ancestral nodes: from 100% probability (black) to 0% (white); probabilities not determined in grey. Divergence times (bottom) are shown in Million years ago (Ma) [42,43].

**Figure 5:**
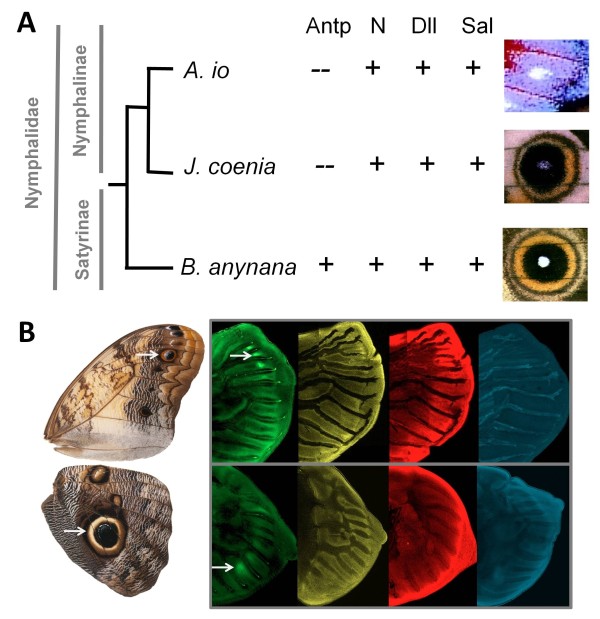
**Divergent positions, morphologies, and underlying gene expression of eyespots**. Examples of inconsistent information from adult phenotype and wing disc gene expression data for homology inference: (**A**) same genetic circuitry and position, yet different morphologies (*J. coenia *and *A. io*); same position and morphology, yet different underlying genetic circuitry (*J. coenia *and *B. anynana*); and (**B**) same genetic circuitry and morphology in eyespots at different positions in the wing (*C. memnon *eyespots in the forewing and proximal spot in the hindwing).

To investigate the evolutionary history of the co-option of conserved genes to the location of a developing novel trait, we analyzed expression patterns in larval wings of multiple species in different butterfly families. We targeted four genes involved in transcription regulation: transcription factors Antp, Dll, and Sal, and the transmembrane receptor N. The latter, when bound to its ligands (Delta/Serrate/LAG-2 family of proteins), releases an Intracellular domain that regulates gene expression when associated to DNA-binding CSL proteins [35]. The expression patterns of *Antp, N*, and *Dll *were previously analyzed across all stages of last-instar larval wings in nymphalids of subfamilies Nymphalinae and Satyrinae [9]. In this study, we added the expression analysis of *Sal *for those same species (Figure 1), and extended the phylogenetic sampling for all four genes to an outgroup comprised of another nymphalid subfamily (Danainae) and two other butterfly families (Pieridae and Papilionidae; Figure 2). Based on the complete dataset for all four proteins in the 13 representative species (Figure 3), we investigated the evolutionary history of the recruitment of these genes. We mapped the localization of transcription regulators in presumptive eyespot centers onto the species tree, and performed ancestral character reconstructions using both parsimony and maximum likelihood (ML) methods (Figure 4). The species chosen in this study represent diversity in (eye)spot morphology and position on the wing (*cf*. the conserved venation pattern), allowing for discussions about the inference of homology (Figure 5).

### Taxonomically wide sampling of genes expressed in the developing eyespot field

In a recent study, we showed that the homeobox transcription factor Antp is found in the presumptive eyespot organizers before Dll and N [9], both of which had, in turn, been characterized as the earliest gene to be expressed in those cells [25,36]. Antp was found exclusively in eyespot centers, whereas N and Dll were also detected in other cells of the wing disc of different butterfly species [9]. Here we add the analysis of expression of another conserved transcription factor previously associated to eyespot development in selected species [30], Sal. We found Sal protein in late larval wings at around the same developmental stage as N [9,25], at the location of future organizers and at the intervein region, consistent with what has been described for *Junonia coenia *[37]. In total, we found the transcription factor Sal in the location of border ocelli pattern elements in five out of ten nymphalid species, in both Nymphalinae and Satyrinae subfamilies (Figure 1).

*Antp *expression during early organizer establishment clearly distinguished Satyrinae and Nymphalinae clades, being present only in the former [9]. In contrast, *N *and *Dll *showed no clear dichotomy between those clades, being expressed in association to most, but not all, developing organizers [9]. Our new data on *Sal *show that its expression is also variable within nymphalids, in a pattern which does not follow that of the other genes (see discussion about gene co-recruitment below) nor that of any particular aspect of eyespot morphology, such as the size, color, shape, or number of rings (see Figure 3).

To infer the evolutionary history of gene recruitment to presumptive eyespot centers, we examined the expression of the four selected genes in a more distantly related nymphalid (*Danaus plexippus*) and in three non-nymphalid species (*Pieris rapae, Parnassius apollo*, and *Papilio machaon*). The monarch butterfly, *D. plexippus*, has series of white spots along the antero-posterior margin of its wings. These appear as multiple single-color spots on each wing compartment bordered by veins, instead of one single element with multiple concentric rings as is characteristic of nymphalid border ocelli (Figures 2 and 3). These single-color spots are generally not considered homologous to border ocelli [36], even though wing patterns of the Danainae subfamily can be described in terms of the Nymphalid Groundplan [20]. On the other hand, many non-nymphalid species have diverse types of spot-like elements that diverge to different degrees from typical eyespots both in morphology (*e.g*. in the number and color of rings) and position; illustrated here by *P. rapae*'s single black spot, *P. machaon*'s quasi-concentric rings, and *P. apollo*'s concentric rings around a white center (Figures 2 and 3) [23]. Whether (see [24] for Papilionidae) or not (see [20,24,30] for Pieridae) these circular pattern elements are homologous to nymphalid border ocelli is unclear. Moreover, little is known about which developmental processes and genes underlie the formation of these different types of patterns. Here we show that none of four transcription regulators associated to eyespot organizers in nymphalids localizes to the regions of the presumptive eyespot-like elements in the outgroup species (Figure 2). Also, with the exception of Dll for *P. rapae*, we could not detect any of those proteins at the intervein region, where N and Dll are found in some butterfly species [25,30].

The absence of all four transcription regulators analyzed from the position of presumptive eyespots in the outgroup species suggests that different mechanisms might be at play in the formation of their spots, as previously suggested for *P. rapae *[30]. Possible scenarios include that 1) the same genes are associated with presumptive organizers but at a stage other than the last larval instar which we analyzed, when nymphalids specify their organizers [33], or 2) other genes are specifying organizers in different butterfly clades, or 3) the spots in these lineages are formed by developmental mechanisms that do not involve central organizers. The latter possibility could be experimentally tested by the same type of tissue transplant or damage approaches that established nymphalid eyespot centers as organizers [39,40], in which the transplantation of such cells to other competent regions of the wing lead to the production of an ectopic eyespot at the host site.

### Ancestral reconstruction of gene recruitment

We coded the localization of the four targeted proteins at the presumptive (eye)spot centers as present or absent for the 13 species (Figure 3), and mapped these characters onto the phylogeny of those species [41-43] by parsimony and ML methods. Regardless of the function of each protein in eyespot formation, their localization in putative (eye)spot centers of larval wing discs can be treated as a character. Mapping this information onto the species tree allows for the inference of the evolutionary history of gene recruitment to that location. Ancestral character reconstructions with both methods showed an unambiguous evolutionary history only for the expression of *Antp *(Figure 4), found at the location of presumptive eyespot organizers of satyrines but not nymphalines [9]. Our sampling of outgroup species supports that the novel *Antp *expression is in fact exclusive to satyrines and originated in the common ancestor of the group (Figure 4).

Ancestral reconstructions of the recruitment of other three transcription regulators resulted in an evolutionary history that is less clear. There are two equally parsimonious scenarios of losses (Figure 4A top) and gains (Figure 4A bottom) of eyespot-related expression for each of those genes, with many instances of homoplastic events. This ambiguity is mainly due to the character states of *Caligo memnon *(absence of N, Dll, and Sal) and *Melitaea cinxia *(absence of N and Sal) in relation to all other members of their respective subfamilies (presence of N, Dll, and Sal). Given the phylogenetic positions of these species, it is not possible to recover a single scenario for the recruitment of the three transcription regulators to the presumptive eyespot organizers. Worthy of special attention is the case of the satyrine *C. memnon*, in which only *Antp *is expressed in the area of presumptive eyespot centers (Figure 3). The forewing eyespot of this species is composed of rings of different colors and placed at the typical location of Nymphalid Groundplan's border ocelli (Figure 1). According to the parsimony reconstructions, either *C. memnon *represents a secondary loss of *N *and *Dll *expression (Figure 4A, upper tree), or the absence of expression of these genes, together with that of *Sal*, is the ancestral state for satyrines (Figure 4A, lower tree). Even though our results do not favor one parsimony reconstruction over the other, the multiple origins or occasional losses of each character state compel us to speculate on the mechanisms by which gene recruitment evolves. For example, how would *C. memnon *have lost expression of both *Dll *and *Sal *(Figure 4A top)? Alternatively, how would *J. coenia *and *A. io *convergently have gained expression of *N *and *Sal *(Figure 4A bottom)? The level of homoplasy found in both parsimony reconstructions might indicate that gene recruitment is a flexible process, whose origin and evolution possibly require minimal changes at key nodes of conserved developmental networks (see [44]). Nonetheless, the expression patterns found in *C. memnon *- a member of the tribe Brassolini, which diverged from the remaining members of the clade (tribe Satyrini) some 60 Million years ago (Ma, Figure 4B and [41]) - uncover variation in which transcription regulators are associated to eyespot organizer regions at the level of tribes.

The ambiguity between the parsimony reconstructions is also reflected in the ancestral state inference obtained with the ML analysis, which estimates with equal probability the presence and absence for each of the three proteins in the eyespot field at the ancestral node of Nymphalidae (Figure 4B). The variable expression found for these genes could only be revealed by having a taxonomically and genetically wide sampling such as we have (see [13]). However, and even though this is the largest comparative study of gene expression patterns in butterfly wing discs available, the evolutionary history of *N, Dll*, and *Sal *expression in eyespot organizer regions requires examination of further species, especially of different subfamilies.

### Evolutionary history of gene co-recruitment

So far, we have analyzed differences in the expression of individual genes in the presumptive (eye)spot centers, and shown that it varies substantially, even within butterfly subfamilies. When we consider the evolutionary history of two or more of the targeted genes together (see Figure 3), we cannot see a consistent co-expression history, which would possibly be indicative of co-recruitment. The only consistent patterns we found were that whenever there is *N *expression, Dll is also present (but not the other way around, see *M. cinxia *in Figure 3) and, whenever there is *Sal *expression, N is also present (but not the other way around, see *M. galathea *in Figure 3). Pairwise comparisons of evolutionary histories, as analyzed by BayesTraits (see Methods section), showed significant correlations for the recruitment of *N*- *Dll *and *N*- *Sal *(Likelihood Ratios of 11.84 and 11.40, respectively, each with *P *= 0.02 for the *χ*^2 ^test).

The four proteins targeted in this study are known to interact in other developmental contexts. For example, Antp activation of N signaling induces *Dll *expression and produces ectopic legs in *Drosophila melanogaster *heads [45]. Earlier in *D. melanogaster *development, Antp promotes the mesothoracic identity of the embryo by repressing *Sal *expression [46,47]. In the presumptive nymphalid eyespot organizers, different combinations of those proteins are found in different species (Figure 3): 1) Antp + N + Dll + Sal, as in *Heteropsis iboina, Bicyclus anynana*, and *Pararge aegeria*, 2) N + Dll without Antp, as in *J. coenia *and *Aglais io*, and 3) Antp without any of the other three proteins, as in *C. memnon*. These different combinations are consistent with either of two scenarios: different proteins were recruited individually to the eyespot field and possibly re-wired *de novo*, or an ancestral network was co-opted and then diversified independently in several lineages (see [12]) possibly involving "partial co-option" (as suggested for abdominal appendages of sepsid flies [48], and for beetle horns [10]).

Another example of co-option of key genes in butterfly eyespots relates to the recruitment of Hedgehog (Hh) [29]. The co-option of Hh was suggested as having led to novel expression patterns of its downstream targets *Patched *(*Ptc*), *Cubitus interruptus *(*Ci*), and *Engrailed *(*En*) in butterfly wing discs [3]. An important finding, however, was that although *Ci *and *En *are expressed in the presumptive eyespots of *J. coenia *and *B. anynana*'s larval wing discs [29], expression of *Hh *and its receptor *Ptc *were never found in *B. anynana *[9]. In other words, shared downstream targets of the Hh signaling pathway are found in presumptive eyespot centers with and without the upstream signal (see also [34]). The differences between those two laboratory models, together with the variation in gene combinations found here for a large number of species, reiterate the suggestion that gene recruitment and co-recruitment is a flexible process. Flexibility in the (co-)recruitment of conserved genes has been found for a few other model novel traits [*e.g*. [10,48,49], but we do not yet know whether it is more probable to gain or to lose expression, whether it depends on particular properties of developmental networks (see [44]), nor which are the more general constraints underlying genetic co-option and its evolution.

### Variation in gene expression and in adult phenotype

The great flexibility found in the (co-)recruitment of the four proteins analyzed to the eyespot fields possibly reflects variation in eyespot development. In examining how these putative recruited or rebuild pathways relate to trait diversification, we observed that the variation of individual or groups of genes targeted in this study does not correlate to any particular aspect of (eye)spot morphology (*e.g*. presence and number of concentric rings) or position (*e.g*. distally located, as is characteristic of nymphalid border ocelli, or in other regions of the wing, see Figure 3). While (eye)spot position is likely established in larval wings where organizing centers are specified [38,50], the color and size of the rings produced around organizers are determined later, in pupal wings [31,32]. A comparative study of transcription factor localization in eyespot fields at this later stage has also reported great flexibility in the association between combinations of transcription factors and the color of nymphalid eyespot rings [31].

When looking at the association between circular pattern elements and the proteins putatively associated with their development, we observed that eyespot morphology, position, and underlying gene expression include three types of potentially conflicting messages (Figure 5). First, eyespots with very similar morphologies and located at the same position in the wing can be found with different combinations of proteins (*e.g. J. coenia *versus *B. anynana*, Figure 5A). Second, very different eyespot morphologies are found even when the same genes are expressed at the same position in the developing wing (*e.g. J. coenia *versus *A. io*, Figure 5A). Third, similar eyespot morphologies with the same gene expression are also found in spots at different positions (*e.g*. the marginal eyespot in the forewing of *C. memnon*, presumably corresponding to border ocelli in the Nymphalid Groundplan, versus the more proximal spot on its hindwing, Figure 5B).

Inferring homology depends on establishing phenotypic criteria (like shared morphology and position [51]) that ideally are matched by developmental criteria (such as shared ontogeny and underlying genetic basis) [38,49-55]. Despite the sometimes extreme differences in morphology (*e.g*. the number, size, and color of concentric rings in *J. coenia *versus *A. io*, Figure 5A), all nymphalid eyespots along the distal half of the wing are considered homologous [20,22-25,30,31]. Our data showed that the putative genetic circuitry of nymphalid eyespot organizer specification is highly variable, reflecting that "homologous structures need not be controlled by homologous genes" [55]. There are other examples of homologous characters that diverge in their development or underlying genetics (reviewed in [53]) and show discontinuity in homology inference at different levels of biological organization [51]. This discontinuity is explained by what has been called phenogenetic drift, that is, the "drift in the relationship between genotypes and a given phenotype" [54] (also referred as developmental system drift [56]). At the same time, disparate eyespot phenotypes within nymphalids (including morphology and position) can be found associated to the expression of the same set of proteins in the larval eyespot field (Figure 5). A similar result has been reported for later stages of eyespot development in pupal wings, where the same transcription factors were found in presumably non-homologous spots (at different positions in the wing) of a nymphalid and a saturniid moth [30]. The pattern elements in lepidopteran wings are a good illustration that phenotypic diversity is not necessarily followed by equivalent levels of genetic diversity [10], being sometimes more and sometimes less variable than the underlying patterns of gene expression.

## Conclusions

Our analysis of the evolutionary history of transcription regulators localization in the (eye)spot fields in larval wings of a variety of butterfly species has revealed substantial variation in the expression of *N, Dll*, and *Sal *within nymphalids. It also established a single origin of *Antp *expression at the presumptive organizer in the common ancestor of the Satyrinae clade. Ancestral reconstructions by parsimony and ML methods for all proteins, together with the lack of phylogenetic evidence for their co-recruitment, revealed ambiguity consistent with both divergence of a co-opted network or independent recruitment of individual genes. The variation found from ancient lineage divergences (among families) to more recent ones (among tribes) shows that the evolution of gene expression associated to the development of this novel trait is highly flexible. Additionally, different butterfly clades (*i.e*. Papilionidae, Pieridae, and Nymphalidae) seem to be using different mechanisms to specify the circular patterns on their wings. Butterfly eyespots illustrate that phenotypic similarity is not necessarily paralleled by similarity in which genes are expressed in association with trait development. Conversely, distantly related species might use orthologous genes to produce non-homologous circular pattern elements on their wings. The differences found between phenotypic and genetic evidence underscore the importance of covering phylogenetic diversity in relation to multiple components of potentially co-opted networks to understand the origin and diversification of novel traits.

## Methods

### Biological material

Thirteen species of three butterfly families were assayed in this study. The nymphalid data on Antp, N, and Dll was obtained from [9] (see reference for details of origin and maintenance of larvae). Additional species, stained for all genes, were obtained from the Lagartagis Butterfly House (Lisbon, Portugal) or field caught and kept as follows: *D. plexippus *(room temperature, and natural light (L) and dark (D) cycle, fed on milkweed), *P. rapae *(18/23°C at 6D:18 L, fed on cabbage), *P. apollo *(27°C at 12D:12 L, fed on stonecrop), and *P. machaon *(27°C at 12D:12 L, fed on fennel). The staging of larval wing development of all families was done following the tracheal extension into the vein lacunae (*cf*. [37]).

### Immunohistochemistry

Immunostainings were performed as in [9] using different staged wing discs covering the entire last larval instar. Right fore- and hindwings from single individuals were stained with anti-Antp and anti-Sal antibody, and left fore- and hindwings were stained with anti-N and anti-Dll antibodies. Antibodies have been shown to be cross reactive across insect orders (*e.g*. [57-59]) and arthropods (*e.g*. [60]). The monoclonal mouse anti-Antp 4 C3 [57] (1:50 dilution) and anti-N C17.9 C6 [58] (1:5 dilution) were obtained from the Developmental Studies Hybridoma Bank. The polyclonal rabbit anti-Dll [60] (1:200 dilution), rabbit anti-Sal [59] (1:500 dilution), and guinea pig anti-Sal GP66-2 (1:1000 dilution, used for *P. rapae*) were provided by other labs. Alexa Fluor 488 anti-mouse, Texas Red anti-rabbit, and Alexa Fluor 594 anti-guinea pig (Molecular Probes) were used as secondary antibodies (1:200 dilution). Images were collected on a BioRad MRC 1024 or a Zeiss Imager M1 laser scanning confocal microscope.

### Ancestral character reconstruction and correlation of protein recruitment history

(Eye)spot centers have been shown experimentally to have organizing properties in selected nymphalid lab models [39,40]. We documented localization of the study proteins at the wing regions corresponding to the developing (eye)spot fields, for those and other species (*cf*. larval venation patterns and (eye)spot location on adult wings). The presence (1) or absence (0) of circular expression patterns at this location was scored for *Antp, N, Dll*, and *Sal *(Figure 3). Reconstruction of ancestral states was done using parsimony and ML methods. Parsimony reconstructs the evolutionary history by minimizing the number of evolutionary transitions (from absence to presence of expression, and vice-versa), favoring reversals (ACCTRAN) or parallelisms (DELTRAN) when two equally parsimonious scenarios exist. Parsimony analyses were performed in WinClada [61] using ACCTRAN and DELTRAN tracing options to examine alternative scenarios in the case of ambiguous optimizations. ML estimates the probability of ancestral states given a model of evolution and takes into consideration the age of divergence between clades. Characters were traced onto a phylogenetic tree generated for the species included in this study. The tree topology used for the character mapping and illustrated in all figures is based on [41] for the family Nymphalidae, and on [42,43] for the superfamily Papilionoidea. Branch length estimates were calculated as described in [43]. ML reconstructions were performed in Mesquite 2.74 [62] choosing the Mk1 model [63].

To assess whether there is significant correlation between evolutionary histories of pair of genes, pairwise Likelihood Ratio Tests were performed comparing the likelihood of an independent *versus *a dependent model of evolution [64,65]. The likelihood for each model was calculated with BayesDiscrete in the BayesTraits package [66], using the branch length estimates and character coding as above. The likelihood ratio was calculated as 2[log-likelihood (Dependent Model) - log-likelihood (Independent Model)], and is expected to follow a *χ*^2 ^distribution with four degrees of freedom [64,65].

## Abbreviations

*Antp: Antennapedia; N: Notch; Dll: Distal-less; Sal: Spalt; Hh: Hedgehog; Ptc: Patched; Ci: Cubitus interruptus; En: Engrailed;* ML: Maximum likelihood; Ma: Million years ago 

Species names: *Aglais io, Junonia coenia, Melitaea cinxia* (Nymphalidae, Nymphalinae)*; Caligo memnon, Melanargia galathea, Pararge aegeria, Lasiommata megera, Heteropsis iboina, Bicyclus anynana* (Nymphalidae, Satyrinae); *Danaus plexippus* (Nymphalidae, Danainae); *Pieris rapae* (Pieridae); *Papilio machaon and Parnassius apollo* (Papilionidae). 

## Authors' contributions

LTS coordinated and co-wrote the manuscript. SVS collected the bulk of the expression data, and MAJ collected individuals and data for *Pieris*. PMB and HD provided *Papilio *and *Parnassius *larvae, and participated in early discussions about comparative analysis of eyespot development. LTS, RAK, and NW performed the phylogenetic analyses. SVS and PB designed the study. PB coordinated the study and co-wrote the manuscript. All authors contributed to the analyses, and revised and approved the final manuscript.
